# Supplementary material for: Are carbon emissions trading and green financial instruments synergistic? -Comprehensive quantitative research based on content analysis
Source: PLoS One. 2024 Mar 7;19(3):e0298601. doi: 10.1371/journal.pone.0298601 (PMC10919601; doi:10.1371/journal.pone.0298601)
Supplement: S1 Data — (ZIP) [file pone.0298601.s001.zip › Data/地级市Z_F工作报告.zip!/╣π╢1⁄2/╒┐╜¡╩╨/└┤╫╘╣1⁄2╓┌║┼í╛┬φ┐╦╩2╛▌═°í┐_2010.docx]

各位代表：

　　我代表市人民政府向大会作政府工作报告，请予审议，并请市政协委员和其他列席的同志提出意见。

　　2009年工作回顾

　　2009年是极不平凡的一年，是我市进入新世纪以来面临困难最多的一年，也是我市全力应对国际金融危机冲击、经济社会发展取得显著成绩的一年。一年来，在中共广东省委、省政府和中共湛江市委的正确领导下，在市人大及其常委会的监督和市政协的支持下，市政府坚持以科学发展观统揽全局，认真贯彻落实中央“保增长、保民生、保稳定”和省“三促进一保持”的决策部署，认真贯彻落实省委省政府“粤西两会”精神，按照汪洋书记关于湛江要“倍加努力，逆势崛起”的要求，深入实施《珠江三角洲地区改革发展规划纲要》和“工业立市、港口兴市、生态建市”发展战略，坚定信心，迎难而上，积极作为，团结奋斗，实现了经济逆势较快发展，各项社会民生事业取得新成效，圆满完成了市十二届人大五次会议确定的目标任务。

　　——保增长取得明显成效

　　经济实力增强。完成生产总值1156.17亿元，增长10.6%，14年来首次超过全省平均水平。其中，第一产业增加值238.87亿元，增长5.5%；第二产业增加值498.79亿元，增长10.8%；第三产业增加值418.51亿元，增长13.2%，对GDP增长的贡献率首次超过第二产业。三次产业比重为20.7∶43.1∶36.2，经济结构进一步优化。人均生产总值16639元，增长9.2%。单位生产总值能耗下降3%，完成年度节能减排指标。地方财政一般预算收入52.65亿元，增长15.2%。

　　工业增势向好。规模以上工业完成总产值1031.46亿元，增长6.1%；完成增加值347.9亿元，增长10.1%。广钢环保迁建湛江项目完成征地拆迁；中科合资广东炼化一体化项目选址湛江，前期工作全面展开；东兴炼油500万吨配套完善、龙腾物流500万吨球团等项目竣工投产；70万吨晨鸣林浆纸一体化项目动工。规模以上工业经济效益综合指数285.56%，居全省前列；实现利税142.44亿元，增长69.4%，其中实现利润79.58亿元，增长97.5%。

　　农业稳中有进。完成农业总产值382亿元，增长6.6％。建立省级现代农业园区6个，南国花卉科技园入园企业52家，新增省级农业龙头企业4家和省农业名牌产品8个、绿色食品认证7个。建设现代标准农田43宗、14万亩，新增节水设施农业面积3万亩，连续10年实现耕地占补平衡。31宗城乡水利防灾减灾工程全面开工，雷州青年运河灌区节水改造工程顺利推进；硇洲、乌石中心渔港建设进展顺利。完成20个建制镇总体规划修编和800条村庄规划编制。农村公路路面硬化1300公里，通行政村公路硬化率99.8%，行政村客运班车通达率90%。开通农业信息联播和“农情气象”业务。建立国家级有害生物预警站。完成造林24.1万亩，森林覆盖率达到28.1%，坡头区创建林业生态县（市、区）通过省验收。

　　投资再创新高。动工和续建重点项目39个，完成投资126.27亿元，增长105.8%，是我市“十一五”时期以来投资项目最多、投资额最大的一年。湛江港东海岛港区散货码头等4个项目竣工，茂湛铁路、湛徐高速公路、东海岛疏港公路及跨海大桥和鉴江供水枢纽工程等10个项目动工建设。在重点项目建设强力带动下，完成固定资产投资393.23亿元，增长33.1%。

　　消费拉动增强。实现社会消费品零售总额571.71亿元，增长22.2%，高于全省5.9个百分点。建成城市商业网点11个，新增营业面积3.1万平方米；镇村两级农家店覆盖率分别达到100%和84%，初步形成县级配送中心为龙头、镇村级农家店为网络的农村消费市场体系。商品房实际销售面积增长56.3%，销售额增长116.3%。市区在建高级酒店5家，总投资35亿元。被命名为“中国海鲜美食之都”。全年接待游客1151.15万人次，旅游业总收入53.25亿元，分别增长29.14%和13.2%。全市港口货物吞吐量1.18亿吨，增长13.8%；其中湛江港集团6427万吨，增长7.9%。湛江民航机场旅客吞吐量39.6万人次，增长30.9%。

　　“双转移”有力推进。编制《湛江市承接产业转移规划》，制定实施《加快承接产业转移的实施意见》。成功竞得全省第五批产业转移示范园扶持资金5亿元。承接产业转移项目25个，投资额89亿元，累计承接产业转移项目205个，投资额460亿元。完成农村劳动力免费技能培训5.1万人，转移就业16.7万人。

　　外经贸逐步回升。受外需萎缩的影响，全市进出口总额28.18亿美元，下降15.3%；其中出口13.65亿美元，下降16.3%，全年降幅比前三季度分别收窄10.3、15.2和6.1个百分点。实际利用外资2925万美元。与南非伊莱姆比地区缔结为友好城市。开展湛江保税物流中心申报工作。

　　体制机制不断完善。如期完成年度国有企业改革任务。调整市对区财政体制，建成财政支出管理电子平台，实现部门预算、预算指标与国库拨款无缝衔接。开展财政投资评审改革和政府投资工程跟踪审计。积极申报设立“广东（湛江）统筹城乡发展金融改革创新综合试验区”；市商业银行广州分行开业；9家农村信用联合社完成统一法人工作；金融机构各项存款余额1316亿元，增长22.34%，贷款余额629亿元，增长32.04%。建立全省地级市首家土地网上公开交易系统，收储土地9171亩，增长2.5倍，实现土地收益9.6亿元。农村土地承包经营权流转有序推进，流转土地124.4万亩。集体林权制度改革和华侨农场体制改革全面推进。开征污水处理和粉尘排污费，适度调整市区生活供水、供气价格，开展全国医药价格改革试点。基本完成市政府机构改革，建立湛江经济技术开发区和东海岛经济开发试验区一体化管理新体制。

　　城市和环境生态建设有新进步。完成《湛江生态市建设规划》和城市综合交通、市区公交、全市土地利用、东海岛新城及石化产业园区等规划编制，开展《“一湾两岸”城市景观规划》编制和城区11个控制性详细规划修编。《湛江市土地利用总体规划大纲》获国土资源部批准。市区建成北站路、站前路，完成人民大道改造；霞山区汉口路、洪屋路片区拆迁改造有新进展，赤坎文保北村改造重点项目农民公寓楼动工建设。完成20条小街小巷改造。开通“12319城建服务热线”。全面启动县（市）城区污水处理厂建设，建成赤坎水质净化厂二期工程和吴川市污水处理厂。58条村庄完成生活污水处理和垃圾收集设施建设，200条自然村建成“林业生态文明村”。新增城市绿地面积70万平方米，顺利通过国家园林城市复检，完成省下达的治污减排任务，饮用水源和近岸海域水质达标率保持100%，市区空气环境质量名列全国重点考核城市第二，城市居住环境和投资环境进一步优化，荣获国家住房和城乡建设部中国人居环境范例奖。

　　——保民生取得明显成效

　　15件民生实事全面落实。全年用于社会民生领域的财政投入75.35亿元，比上年增加10.84亿元，占地方财政一般预算支出的61.84%，是社会民生投入力度最大的一年，较好地完成年初确定的15件民生实事。取消43项行政事业性收费，减少企业和群众负担7.2亿元；1000条自然村实现广播电视“村村通”；建成农家书屋216间；新增城镇就业6.5万人；开展创业培训2300人，带动就业1.1万人；城镇失业人员实现再就业5.1万人，其中困难对象就业5157人，城镇登记失业率3.12%；举办206场农民工招聘会；农村家庭年人均纯收入低于1500元的困难户全部纳入低保；养老保险参保62.8万人、失业保险参保33.2万人、城镇职工医疗保险参保47.3万人、工伤保险参保26.8万人、生育保险参保33.6万人；建设廉租住房921套、5万平方米，建成620套、3.37万平方米；完成农村困难群众危房改造1.6万户；建成水库移民新村85条，建房45万平方米，解决了3989户、1.75万人的住房困难；农村饮水安全工程开工70宗、竣工46宗，解决103.4万人饮水安全问题；动工建设村镇敬老院123间；全面启动355个贫困行政村脱贫帮扶工作；7家省级扶贫农业龙头企业带动1.75万贫困农户实现增收。市区居民家庭人均可支配收入13665元，增长10.5%，创近5年新高；农村居民人均纯收入5895元，增长10.6%。

　　社会事业全面推进。实施各类科技计划项目220项，其中国家级41项、省级141项；专利申请量和授权量分别增长10%以上；新增各级工程技术研发中心和农业创新中心8家。调整中小学布局，新建扩建学校49所，增加学位3.7万个；高中阶段毛入学率75.8%，比上年提高10个百分点；基本实现中小学教师工资福利待遇“两相当”，1925名代课教师转为公办教师。新增省级非物质文化遗产保护项目6个；一个雷剧剧目获省第七届精神文明“五个一工程”优秀作品奖；文车龙狮团等一批民间艺术团队参与对外文化交流赢得良好声誉；湛江粤剧院加快建设；创建特色文化村294个，建成农村文化室5000多个。率先在全国欠发达地区实现城乡居民基本医疗保险一体化，参保546万人；减免3210名困难群众医药费220万元；医疗机构上网采购率98%以上，减轻群众医药费负担7200多万元；城市社区卫生服务覆盖率100%，行政村卫生站覆盖率95%；有效防控甲型H1N1流感等重大传染病；6个中医名科和特色专科被评为省级重点专科；市一中医院被授予“广东省中医名院”。成功申办2014年省第十四届运动会和举办市第十一届运动会、第十届体育节、第三届国际海上龙舟邀请赛；参加全国第十届运动会取得优异成绩。住房公积金用于职工购房贷款增长135.35%，为保障和扩大住房消费发挥了积极作用。人口出生率11.97％。、自然增长率7.41％。，完成省下达的人口计划指标。对口支援四川省汶川县龙溪乡地震灾区55个援建项目全部开工，34个项目竣工。完成粮食清仓查库。被评为第二次全国经济普查先进单位。荣获省“重大建设项目档案工作质量优秀奖”。实施数字遥测地震台网升级改造和农村民居地震安全示范工程。建成市人防应急机动指挥平台。成功举行军地联合动员支前和防卫作战实兵演习，民兵预备役建设经验在全国推广。建成生态文明村4887个，其中全国文明村6个、省级文明村42个，占全市自然村总数的39%。统计、气象、海防、地方志、社科、民族宗教、妇女儿童、残疾人、老龄、国家安全等工作取得新成绩。

　　——保稳定取得明显成效

　　社会管理水平进一步提高。切实加强信访工作，积极调处化解各类矛盾纠纷，全力预警防控群体性事件，逐一清理积案，有效防控新发案。全面推进镇街综治信访维稳中心基础建设，提前实现省目标。建立市应急管理专家组、专家库和粤西三市应急联动机制，开展应急管理“五个一”示范工程建设。组建消费者维权律师团、消费指导专家团和工会职工维权律师志愿团，维权服务工作站点覆盖全市100%社区和50%乡村，积极发挥农村维稳律师团作用，近2万人次受到法律援助和服务。市区六好平安和谐社区达标125个，占社区总数42%。创建“无传销社区（村）”1175个。严格落实安全生产责任制，实现省下达的安全生产控制目标。坚持打防结合，扎实开展反走私综合治理。组织开展严打专项行动，严厉打击各类犯罪活动，破案率上升25.1%，全市治安保持稳定。

　　市场经济秩序进一步规范。认真落实注册资本“零首期”、登记管理“零收费”、扩大股权出资范围、小额贷款公司试点登记等优惠政策，积极为各类市场主体提供优质服务。民营经济增加值540.72亿元，增长13.4%。物价总体水平基本保持稳定，居民消费价格指数下降0.6%。加大食品、药品、产品质量安全监管、专项整治和执法打假力度，严厉查处经济违法违规案件，有效净化市场环境。

　　民主法制建设进一步加强。不断健全各级政府领导班子议事规则和决策机制，完善基层政权、企事业单位民主管理制度。办理省、市人大代表议案、建议和政协委员提案各157件，办复率均为100%。建立行政审批和重点工作、重点项目电子监察系统，47个部门612件事项进入网上审批，审批事项平均承诺办理时限比平均法定时限缩减64%；工程报建审批效率提高78.9%。规范性文件报备率和及时率达到100%。成功举办“北部湾廉政论坛”。政务公开工作在全省考核中获得优秀成绩。创建民主法治示范村（社区）225个，依法治市和普法工作被评为省先进单位。

　　各位代表，回顾2009年，在国际金融危机不断扩大、国内外经济环境复杂多变、面临异常困难的背景下，我市经济社会保持了平稳较快发展，绝大多数指标超过全省平均水平，其中部分主要指标提前完成“十一五”规划目标任务。成绩来之不易，经验弥足珍贵。回顾一年来的工作，我们从中得到深刻的体会：必须把贯彻落实中央和省委省政府应对国际金融危机的决策部署与创造性开展工作紧密结合起来，做到态度坚决、行动迅速、执行有力，形成抢抓机遇、加快发展的强大向心力、凝聚力和战斗力；必须坚持突出重点项目拉动作用，争分夺秒精心组织重点项目建设，以重点项目建设拉动投资、扩大内需，带动各项工作全面推进，带动促增长保发展各项措施全面落实；必须坚定不移推进以工业为主导的现代产业体系建设，不断做强工业、优化农业、提升服务业，扎实推动产业和劳动力“双转移”，实现三次产业相互促进、互为支撑，推动经济发展方式转变和结构调整；必须矢志不渝保障和改善民生，千方百计解决涉及广大群众切身利益的现实问题，坚持不懈为人民群众办实事办好事，激发社会各界同心同德、共克时艰的积极性主动性；必须大力转变作风狠抓工作落实，始终保持强烈的责任意识和苦干实干作风，勇于担当，敢于负责，富于创新，确保每项工作、每件任务抓早、抓实、抓主动，取得扎实成效。在此，我代表市人民政府，向各级人大代表和政协委员，向全市广大干部群众，向驻湛人民解放军、武警官兵、人民警察和各民主党派、人民团体、社会各界人士，向关心支持湛江发展的港澳台同胞、海外侨胞、国际友人，表示崇高的敬意和衷心的感谢!

　　在充分肯定成绩的同时，我们也清醒地看到，我市经济社会发展与落实科学发展观、建设和谐社会、争当粤西振兴发展龙头的要求，与全市人民的愿望和期待，还有明显差距，还存在不少困难和问题：一是支撑加快发展的产业基础不稳固，工业总量不大、规模较小、集聚度不高，尚未形成具有较强辐射带动力的产业板块和产业集群，先进制造业和高新技术产业发展滞后，现代服务业亟待升级换代；二是城市集聚发展水平不高，综合服务功能不强，辐射带动能力不足，尤其是旧城区和城中村改造进展缓慢，城市管理水平较低，交通秩序和违法违章建筑问题突出；三是区域发展不平衡，县域经济薄弱；四是对外开放水平明显偏低，外贸出口和实际吸收外资总量偏小、降幅较大；五是保障民生任务艰巨，就业、社保、教育、文化、卫生等社会事业发展有待加强；六是政府工作还存在不足，一些部门和干部的思想观念、履职能力、工作作风与科学发展的要求还不适应，等等。对此，我们务必高度重视，采取强有力措施认真解决。

　　2010年工作安排

　　2010年是全面完成“十一五”规划的冲刺之年，也是深入贯彻落实省委省政府“粤西两会”精神，实现“三年大变化、十年大跨越”，争当粤西振兴发展龙头的开局之年。做好今年的政府工作，对夺取应对国际金融危机影响的全面胜利，巩固经济社会平稳较快发展的好势头，为“十二五”规划启动实施、为今后更长时期加快发展打牢基础，具有重大意义。

　　今年政府工作的总体要求是：坚持以科学发展观为指导，认真贯彻党的十七大、十七届三中、四中全会和胡锦涛总书记视察广东重要讲话精神，全面落实中共广东省委十届六次全会、省十一届人大三次会议和“粤西两会”的决策部署，按照“五个更加注重”、“五个扎实推进”的要求，以争当粤西振兴发展龙头为目标，全面实施“工业立市、港口兴市、生态建市”发展战略，大力推进“三促进一保持”，坚定不移调结构，脚踏实地促转变，积极有效扩内需，坚持不懈抓改革，主动有为谋创新，千方百计惠民生，苦干实干抓落实，努力实现经济社会又好又快发展，全面完成“十一五”规划目标任务。

　　今年经济社会发展的主要预期目标是：生产总值增长12%；人均生产总值增长10.8%；全面完成“十一五”节能减排目标责任；工业增加值增长12%；全社会固定资产投资增长20%；港口货物吞吐量1.28亿吨；社会消费品零售总额增长20%；居民消费价格指数涨幅控制在4%左右；外贸进出口总额增长6.5%，其中出口增长8%；实际吸收外资增长20%；地方财政一般预算收入增长11%，人均财政一般预算收入增长9.8%；城镇居民人均可支配收入增长9%，农村居民人均纯收入增长8%；城镇登记失业率控制在3.5%以内；人口自然增长率控制在8％。以内。

　　今年政府工作的重点是：

　　一、着力转方式调结构，加快构建现代产业体系

　　要把转变发展方式、调整经济结构作为主攻方向，努力做强做大以重化工业为引领的现代工业、以港口物流和滨海旅游为引领的现代服务业，重点培育现代产业50强项目，大力推动技术改造和自主创新，构建具有湛江特色优势的现代产业体系。

　　加快建设钢铁石化产业基地。全力推进广钢环保迁建湛江项目，力争一年建成搬迁村民安置小区；扎实做好中科合资广东炼化一体化项目各项前期工作，创造条件尽快动工。编制实施《湛江钢铁产业发展规划》和《钢铁石化循环经济发展规划》，推进建设钢铁、石化主导和配套产业园区，谋划引进一批关联度高、投资量大、带动性强的产业链及配套项目和企业，着重培育装备制造、钢铁冶炼、石化等先进制造业10强项目，打造国家级重化循环经济产业示范基地。

　　加快发展优势特色产业。推进晨鸣70万吨林浆纸一体化和冠豪特种纸项目，扩大利用蔗渣、剑麻纤维等造纸，规划建设制浆造纸产业园，逐步建成广东重要造纸基地。大力发展蔗糖深加工和综合利用，延长产业链，推动糖业向以糖为主、多业并举综合性产业转型。整合资源支持省市共建小家电园区，积极实施绿色环保联盟标准工作，提升产品档次，形成品牌系列，建设具有明显竞争优势的小家电生产基地。推动农海产品生产标准化、加工集约化、经营规模化，打造国家级农产品加工基地。重点培育造纸、农产品加工、电气机械及器材等优势传统产业10强项目。

　　加快提升现代服务业。完善港口货物调运、加工、仓储、分拨、转口贸易等配套设施，大力发展石油、铁矿石、煤炭、粮食、食糖、化肥等临港市场和集装箱运输市场，增加东盟近洋班轮航线和国内外集装箱联运快线，培育临港服务业集聚区。支持驻湛金融机构稳健发展；支持市商业银行改革股权结构，打造地方金融龙头企业；支持外地商业银行设立驻湛机构，积极引进股份制商业银行、金融租赁公司、农业保险公司；探索创办科技银行，加快发展金融产业。推动湛江经济技术开发区旧区、海滨大道和椹川大道沿线工业企业迁入工业园区，重点建设乐山大道两侧、霞海港片和赤坎旧大天然片中央商务区。全面实施《湛江市商业网点发展规划》，完善商贸流通、旅业餐饮、休闲娱乐等基础设施，着力发展以便民利民为主的社区服务业，推进社区服务社会化；建设粤西县域小家电产品及配件批发市场，继续做大汽车销售市场；在主产地或主销区、集散地扶持发展一批水果、蔬菜、海产品等专业批发市场，改造海田批发市场，迁建霞山水产品批发市场，增建和改造市区农贸市场，建设华南食糖物流中心和花卉销售中心。积极扩大“中国海鲜美食之都”品牌效应，编制全市餐饮业发展规划，举办大型海鲜美食节庆活动，进一步繁荣餐饮服务业。支持发展信息、社会中介和会展等服务业。重点培育港口物流、信息服务、房地产等现代服务业10强项目。

　　加快发展旅游业。制定实施《湛江市旅游产业发展规划》，扎实抓好湖光岩五星级温泉度假村、霞山渔人码头及欧陆风情街、特呈岛度假村、吴川吉兆湾海洋休闲度假中心等14个重点项目建设，完善市区主要公园旅游休闲设施，规划建设一批海鲜美食、特色购物、文化娱乐、运动健身等服务项目，打造滨海休闲旅游产业带。支持湖光岩创建国家5A级旅游景区、东海岛旅游区创建国家4A级旅游景区和特呈岛创建国家级滨海生态旅游区。大力开发特色乡村旅游，县（市、区）年内至少建成一个大型“农家乐”旅游项目，形成特色乡村旅游集群。主动对接海南国际旅游岛建设，重点完善徐闻大汉三墩、白沙湾和雷州天成台、雷文化旅游区基础设施，增强对游客吸引力，与海南形成优势互补、错位发展格局，努力构建雷琼旅游产业带。

　　加快培植高新技术产业。深入实施知识产权战略和《湛江市自主创新行动计划》，扎实推进10大科技创新工程、20个科技创新专项和200个市级以上科技计划项目；构筑产学研合作技术创新体系，设立中小企业科技创新基金，推动创新要素向企业集聚，重点培育钢铁石化新材料、生物医药、电子信息、新能源与节能等战略性新兴产业10强项目。申报建立省级高新技术产业开发区。评选公布全市“自主创新50强”企业。

　　加快推进“双转移”。着力推进广州（湛江）产业转移园区建设，用好用活省5亿元扶持资金和1亿元专项资金，完善基础设施，提升园区功能，优化服务环境，强化产业招商，努力建设全省最具发展潜力的“双转移”示范区。坚持园区开发与招商引资、项目建设同步进行，加快龙岗（吴川）、顺德（廉江）产业转移工业园和各类工业集中区建设，做到开发一片，兴建或投产项目一批，实现园区产业集群化发展。支持湛江农垦湖光工业园区和华侨管理区工业园区建设。坚持承接产业转移与扩大农村劳动力转移就业相结合，加强县级劳动力技能培训示范基地建设，深化与珠三角劳务合作，促进农村劳动力向本地重点企业、工业园区和珠三角转移就业。

　　二、着力夯实农业基础，统筹城乡协调发展

　　我市是农业大市，必须把解决“三农”问题摆上重要位置，认真贯彻落实今年中央一号文件，更加注重统筹城乡发展，进一步加大“三农”投入，全面落实强农惠农政策，加快传统农业向现代农业转变，建设农业强市。

　　加强富县强镇建设。扎实推进富县强镇事权改革，扩大县镇经济社会管理自主权，建立促进县镇经济发展的新机制。推动支柱工业尤其是钢铁、石化产业配套项目和能源项目向县域布局，发挥资源优势培育特色产业集群，提升县域工业化水平。支持县（市、区）建设跨区域基础设施，加快打通高速公路出入口与县（市）城区公路及工业园、旅游区连接线，推进县域港口建设。坚持城镇建设与农场场部、工业园区、商住小区和专业市场建设紧密结合，落实中心镇发展优惠政策，以公共服务设施和公共事业建设为突破，增强城镇综合服务功能，落实放宽中小城市和小城镇户籍限制政策，引导农民、资金和产业向城镇集中，扩大城镇规模和人口。

　　加强现代农业建设。编制实施《湛江现代农业发展总体规划》，着力培育建设优质粮、高产高糖甘蔗、无公害蔬菜、特色热带水果、花卉、水产养殖与加工等现代农业10强项目，延伸现代农业产业链。大力推进与泰国正大集团合作建设我市现代农业项目，带动农业资源开发和精深加工，加速资源优势转化为商品优势。深化与湛江农垦全方位合作，推进海峡两岸农业合作试验区和南国花卉科技园建设，打造现代农业示范园区。落实扶持农业龙头企业发展政策措施，支持有比较优势的农业龙头企业加快发展，推动农业产业化经营。积极发展现代渔业，建设国家级现代海洋渔业基地。建成区域性农业综合试验站和国家级农产品质量检测中心。完善农村土地流转市场，建立健全土地流转服务组织、服务网络和激励机制，促进土地适度规模经营。积极稳妥推进集体林权制度改革。扶持发展农村集体经济组织、农民专业合作社和农村经纪人，完善农村专业化、市场化和社会化服务体系。

　　加强农业基础设施建设。加快雷州青年运河灌区节水改造工程；完成7宗大中型、111宗小型病险水库除险加固和31宗城乡水利防灾减灾工程。推进农田基本建设，实施农田综合开发土地整理项目13.1万亩，补充耕地5万亩。完善硇洲、乌石国家级中心渔港基础设施，申报建设龙头沙、草潭国家一级渔港。

　　加强新农村建设。坚持把生态文明村创建活动贯穿于新农村建设全过程，以村镇规划为引领，以“万村百镇”整治工程为载体，以“四通五改六进村”为主要内容，扩大生态文明村覆盖面。年内完成25个建制镇总体规划修编和600条村庄规划编制。全面完成农村饮水安全、安居和道路畅通工程，协调解决库区移民供电问题，建成通自然村硬化道路800公里，启动50条村庄简易生态污水处理和垃圾收集设施建设试点。继续抓好首批35个生态文明区（片、带）建设和200条自然村“林业生态文明村”创建活动，选择5个镇和20条村庄开展宜居城乡试点，在有条件的地区整镇推进，推动生态文明村成片集群发展。

　　三、着力扩大内需，增强发展动力

　　坚定不移地贯彻实施扩大内需战略，努力促进投资稳定增长，积极扩大消费需求，不断增强经济发展的自主性、协调性和可持续性。

　　大力推进重点项目建设。今年计划安排重点建设项目62项，总投资1316.54亿元（不含前期预备项目），年度计划投资215.58亿元；其中列入省重点项目34项，年度计划投资177.88亿元。年内建成湛徐高速公路、东海岛疏港公路及跨海大桥一期工程和国道325线文车至北罗坑段一级公路；加快湛江港30万吨级原油码头、宝满港区集装箱码头一期、霞山港区30万吨级散货码头、茂湛铁路、海湾大桥连接线二期、南三大桥、鉴江供水枢纽工程和奥里油电厂油改煤、湛江国家石油储备库、粤电徐闻风电场等项目建设；动工建设东海岛铁路、东海岛跨海大桥二期和霞山海滨码头改扩建工程；启动湛江港40万吨级航道、东海岛至南三岛海底隧道、东海岛至雷州跨海通道、东海岛石化园区通用码头、大唐国际雷州发电和中海油粤西LNG接收站等项目前期工作。

　　大力促进民间投资。全面落实促进民营经济发展的政策措施，放宽市场准入条件，凡符合国家法律及产业政策的行业和领域，都向民营资本开放，积极促进民营资本参与基础设施、公共服务和社会事业建设。实行民营、国营平等的收费和价格政策。支持湛江籍企业家回乡创业。设立民营创业投资引导基金，加大贷款贴息等财政扶持，开展中小企业融资租赁业务试点，完善信用担保和社会化服务体系，为民营投资创造优良环境。

　　大力扩大消费需求。积极培育和营造消费新热点，进一步扩大汽车、教育培训、旅游休闲、家政服务、文化娱乐、体育健身、信息服务等新型消费。认真落实国家促进房地产市场健康发展政策，增加中小户型、中低价位普通商品房用地供应，引导房地产企业推出更多符合市场需求的产品，规范活跃二手住房交易和租赁市场，充分用好住房公积金，提高干部职工购建住房能力，支持农民依法依规建设自用住房，扩大住房消费。实施国民旅游休闲计划，落实带薪休假和弹性休假制度，发行旅游消费券，扩大旅游消费。推进万村千乡、双百市场和农超对接工程，继续开展家电、汽车、农机、建材下乡和汽车、家电以旧换新活动，扩大农村消费。支持各级消费者委员会加强消费维权工作。组织参与“广货北上”、“广货全国行”等活动。

　　四、着力加快中心城市建设，提升综合服务功能

　　争当粤西振兴发展龙头，必须坚持以产业和人口集聚为重点，按照建成全国重要的沿海开放城市、现代化新兴港口工业城市、生态型海湾城市、粤西地区中心城市和环北部湾重要城市的目标，加快中心城市发展，不断提升辐射服务功能。

　　高起点抓好发展规划。高标准、高质量完成“十二五”规划编制工作。组织编制《湛江市城乡发展战略规划》、《湛江巿城市总体规划》和《湛江市2011-2015年城市近期建设规划》，完成“一湾两岸”城市景观规划，科学合理布局城市组群，努力打造众星拱月、拥湾发展的生态型海湾城市。配合省编制粤西地区城镇群协调发展规划，建立区域规划合作协调机制。编制湖光农场片分区规划。

　　加快完善市政基础设施。结合城市防洪工程重点开发滨海区域，建设东海岸观海长廊和主干道网络，高标准开发海东新区。建成海淀路、奋勇大道、体育北路西段和海田路景观整治二期工程，基本完成南方六横路、华田路、明政路等8条骨干道路建设和滨湖路整治二期工程，推进疏港大道二期工程和海滨大道改造，启动湛江大道、新湖大道、金沙湾观海长廊三期工程。建设东海岛和霞山地表自来水厂，扩建麻章自来水厂。完成编制市区公交发展规划，改造海田公交总站、霞山汽车南站和麻章公交站，增建LNG加气站场。

　　加大“三旧”改造力度。制定市区“三旧”改造规划和实施方案，用足用活省“三旧”改造政策。完成霞山水产品批发市场搬迁，加快汉口路、洪屋路、东堤路片和旧大天然片、霞海港片旧城区集中连片改造，打造城市精品片区；有序推进城区九个重要节点“三旧”改造，全面提升城市环境品质和景观风貌。抓好文保村整村改造试点，以点带面，按照居住条件完善、公共服务配套、人居环境优美的要求，逐步把城中村改造成优美的城市宜居社区。

　　加强环境生态建设。全面实施《湛江生态市建设规划》，以海湾生态保护为核心，加快建设沿海防护林、交通绿色长廊、江河水源涵养林等七大生态带；以保护饮用水源为重点，强化主要河流环境监测整治和城市水体治污。推进海洋保护示范区建设，坚决治理工业及船舶油污、海岸虾池、网箱养殖等污染源。扎实推进国家卫生城市、国家环保模范城市、国家生态园林城市和广东省林业生态县（市、区）创建活动。坚决落实节能减排问责制，确保完成“十一五”节能减排目标。深入实施城乡清洁工程，建成坡头区、东海岛和各县（市）城区污水处理厂，动工建设市区无害化生活垃圾处理场三期填埋区和生活垃圾焚烧发电厂，完成赤坎江二期和南柳河东新渠整治。继续实施公共交通清洁能源改造，逐步实现市区公交车全部改用天然气。加快主要公路绿道的规划改造，启动城市绿道和社区绿道建设，建成滨湖公园一期工程，扩大和改造三岭山森林公园，启动建设银帆公园、麻章公园和东海公园，年内市区新增绿地面积100万平方米以上。完成造林20万亩，全市森林覆盖率达到28.2%。

　　提高城市管理水平。推进城市管理信息化，完善数字化城市管理系统平台，建立数字化管理与部门监管相结合、区负责与市协调、监督、考核相结合的城市管理体制，将城市管理执法重心下移到区、街道，实现“一级执法、二级管理、责任在基层”。充分用好城管热线，引导市民广泛参与城市管理。积极探索运用市场机制推进城市管理的途径和办法。强力整治城区交通秩序，突出解决无牌无证车辆上路和交通拥堵问题。加强城乡结合部规划管理，坚决整治和遏制违法违章建筑。

　　五、着力深化改革开放，擦亮沿海开放城市品牌

　　改革开放是强市之路。必须不失时机推进重点领域和关键环节改革，不断提高对外开放水平，为争当粤西振兴发展龙头不断注入新的动力和活力。

　　深化国有资产管理和国有企业改革。完善国有企业法人治理结构和出资人制度，推进事业单位财务预算管理试点，扩大行政事业单位经营性资产委托管理范围。积极推动市属国有企业与中央和省属国有企业合资合作，引入社会资本实行公司股份制改革，推进产权多元化。支持有实力国有企业“走出去”实现更大范围资源优化配置。引入战略投资者和民间资本盘活国有企业有效资产。稳妥有序引导30家劣势企业退出市场。加快华侨农场行政体制改革。全面实施事业单位人事制度改革和岗位设置、人员公开招聘工作。

　　推进基本公共服务均等化。落实《广东省基本公共服务均等化规划纲要》，优化财政支出结构，加大公共支出；探索基本公共服务多样化供给形式，鼓励和引导社会组织或个人参与公益服务，逐步形成惠及全民的基本公共服务体系。健全市、县（市、区）、乡镇三级财权与事权相匹配的财政体系，完善市对区财政管理体制，积极做好省直管县财政对接工作。扩大乡镇财政和村级财务管理方式改革试点。加强税收征管。

　　创新投融资体制。推进“广东（湛江）统筹城乡发展金融改革创新综合试验区”建设，争取国家和省相关金融政策先行先试，进一步优化区域金融生态环境。支持湛江港集团等优势骨干企业上市融资。组建市基础设施建设投资集团、市交通基础设施建设投资集团、东海岛综合开发投资集团、市水务集团四大投融资平台公司，运用市场机制拓宽融资渠道，广泛吸纳各类资本投入城市和基础设施建设。推进9家县级统一法人农村信用联合社逐步改制为农村商业银行或合作银行，鼓励发展融资性担保公司，开展村镇银行和小额贷款公司试点工作，争取每个县（市、区）建立一家以上小额贷款公司。

　　力促对外贸易稳定增长。建立市、县（市）两级外经贸财政扶持机制，支持企业开展出口信保业务。创建出口自主品牌，优化进出口商品结构，推动传统出口产品升级换代，扶持优势产品和企业申报国家、省级出口名牌，推动有规模、有优势的加工贸易企业向自主设计、自主品牌转型，提高出口市场竞争力。抓住中国-东盟自由贸易区正式建立的机遇，主动开拓东盟市场。积极策划和组织参加境内外各类展会及贸易投资推介活动，抢抓客户和订单，扩大产品进出口。完成口岸整合，推进电子口岸三期工程，加快东海岛港区、霞山宝满港区等口岸查验配套设施建设，改善口岸通关环境。积极应对国际贸易壁垒，支持企业维护合法权益。抓好申报设立保税物流中心工作。

　　扩大对外招商引资。按照“招大、引强、选优”的要求，精心谋划推出一批重大对外招商项目，紧紧围绕钢铁、石化、家电、农海产品加工等主导产业开展产业链招商，培育外向型经济龙头。充分发挥我市作为广东进军东盟海上“桥头堡”优势，积极实施“走出去”战略，扩大对东盟招商。进一步强化湛江经济技术开发区在全市招商工作中的龙头带动作用。积极主动做好签约项目跟踪落实工作，对增资扩产企业和项目加强服务，力争更多外资到位。申办2010年广东省首届“海博会”。

　　加强区域经济合作。认真落实《阳江、湛江、茂名、云浮四市经济社会发展紧密协作框架协议》，积极推动粤西一体化发展。主动融入珠三角经济圈，建设服务珠三角制造业的原材料基地。争取纳入北部湾经济区发展规划，制定实施与广西北部湾经济区和海南国际旅游岛发展规划相配套的高层次发展战略，努力实现优势互补、共同发展。进一步发挥大西南出海主通道优势，重点加强在港口物流和转口贸易等领域合作，培育区域大物流圈。积极开展东盟区域友好城市结交工作，扩大与国内外友好城市经贸合作成效。

　　六、着力改善民生，构建和谐湛江

　　始终把保障和改善民生作为发展的最高目标，努力实现好、发展好、维护好人民群众的根本利益，使广大群众分享更多发展成果，感受更多公平正义，体验更多和谐幸福。

　　积极促进充分就业。继续完善和实施积极就业政策，坚持就业惠民、创业富民导向，形成以创业带动就业、创业就业带动扩大内需的良性循环。建立健全公共投资促进就业增长机制，加强创业项目开发，推进创业型城镇和创业示范基地建设。认真落实鼓励企业增加就业岗位、加强就业培训和减负稳岗的财税、信贷、价格优惠政策。支持高校毕业生到中小企业、城乡基层就业。完善就业援助制度，帮扶就业困难对象和零就业家庭成员就业。

　　提高社会保障水平。完善多层次养老保障体系，努力解决城镇无保障居民养老、困难企业退休人员和职工医疗保障问题。进一步提高社会保险费地税全责征收水平和保障能力，扩大受惠面。逐步提高城乡低保标准，按期调整企业退休人员养老待遇。实行养老保险关系跨省市无障碍转移。积极推进农村新型养老保险试点，落实困难群体参保补助政策。实施被征地农民养老保险制度，做到“应保尽保、即征即保”。不断提高城乡医疗保障覆盖率，探索构建职工医保和城乡居民医保统一管理、可转换衔接的基本医疗保险制度。完善失业保险政策，开展失业保险基金扩大支出范围试点，建立集保障生活、预防失业、促进就业三位一体的失业保险体系。

　　优先发展教育事业。建立县域义务教育资源合理配置机制。以集聚办学为主要内容，推进学校布局调整优化，促进义务教育均衡发展。继续实施中小学校舍安全工程，完成规划改造任务。推进高中学校建设，确保完成普及高中阶段教育任务。加快建设职业教育基地。支持广东医学院建设新校区、广东海洋大学建设海洋科技产业园（东海岛基地）和湛江师范学院扩大规模。继续抓好中小学代课教师“民转公”和落实教师绩效工资工作。促进发展民办教育。

　　壮大医疗卫生事业。深化医药卫生体制和价格改革，开展公立医院改革试点。支持迁建湛江中心人民医院，扩建广东医学院附属医院，建设市传染病医院、湛江中心人民医院东海岛分院和市紧急医疗救援指挥中心。推进医院信息化和城市社区卫生服务设施建设。进一步健全以县级医院为龙头、乡镇卫生院和村卫生站为基础的农村医疗卫生服务网络。鼓励发展民营医疗机构。加强甲型H1N1流感等重大疾病防控。促进中医药强市建设。

　　建设文化体育强市。广泛开展“四德五心六歌”活动，着力构建社会主义核心价值体系。大力推动石狗、醒狮、飘色、人龙舞等特色历史民俗文化走出湛江，打响雷州国家历史文化名城品牌。继续实施广播电视“村村通”、文化信息资源共享、农村电影放映、非物质文化遗产保护和农家书屋建设五大工程。开展“文化遗产日”、“国际博物馆日”、“世界读书日”、“全民读书月”及“图书馆服务宣传周”活动。抓好第三次全国文物普查。深化文化体制改革，完善政府引导、市场运作的文化发展机制，引导各类资本进入文化市场，加快发展文化产业。推进建设湛江粤剧院和新闻大厦。开展文化艺术中心前期工作。启动建设市新档案馆，完成档案事业发展综合评估和新农村档案示范点创建工作。加快省第十四届运动会体育场馆建设，加强竞技体育后备人才培养，组织参加省第十三届运动会，办好第四届中国（湛江）海上国际龙舟邀请赛、市第十一届体育节和市第四届社区运动会。

　　加强扶贫开发工作。完善扶贫开发“规划到户、责任到人”工作机制，全面推进产业化扶贫、基础设施扶贫、转移就业扶贫、科技扶贫和智力扶贫，重点抓好355条贫困村脱贫工作，为完成三年预期目标打下坚实基础。设立“扶贫济困日”，鼓励对口帮扶单位及社会各界深入贫困村、贫困户开展多种形式扶贫活动。做好四川省汶川县龙溪乡地震灾区援建期帮扶工作。

　　努力建设平安湛江。深入推进普法和依法治市工作。进一步完善应急管理和救援体系，提高应对突发事件能力。健全矛盾纠纷排查、预警、化解和处置机制，积极发挥各级综治信访维稳中心作用，年内建成市、县两级人民来访接待厅，坚持领导干部基层大接访和包案制度，妥善处理各方面利益关系和解决热点难点问题。落实人口计生目标管理责任制，统筹人口发展。加强国防后备力量特别是基层民兵预备役“四个基本”建设，深入开展军政军民双拥共建工作。切实抓好统计、人力资源、外事、侨务、民族宗教、对台、人防、地震、修志、老区、粮食、供销社、气象、社会科学以及妇女儿童、残疾人、老龄、殡改等工作，促进各项社会事业协调发展。创新食品药品安全联合监管模式，确保人民群众生活和用药安全。强化市场价格监测和综合调控，推进资源环保价格改革，保持物价总体水平基本稳定。加强文化市场综合执法管理，加大打击网吧违规经营力度。全面落实安全生产责任制，强化安全生产基层、基础管理；加大排查和治理安全生产隐患力度，加强消防设施和消防队伍建设，确保完成安全生产控制指标。进一步抓好群防群治工作，积极探索治安管理与城市管理、市场管理、行业管理有机结合的新模式，建立健全城乡一体、内外协调、技术兼容、资源共享、专群结合的治安防控网络；始终保持打击走私高压态势，完善反恐工作机制；坚决整治社会治安突出问题，依法严厉打击严重刑事犯罪尤其是涉黑、涉枪、涉抢、涉毒等犯罪，促进社会治安根本好转。

　　办好18件民生实事。改造市区88条小街小巷；安装市区61公里3000盏路灯；新增城镇就业5万人；完成农村劳动力技能培训4万人，新增转移就业15万人；全额资助17万低保对象和5.4万五保户免费参加城乡基本医疗保险；把大中专在校学生纳入城乡居民医保范围；年内养老保险参保达到65万人、失业保险参保达到34万人、城镇职工医疗保险参保达到48万人、工伤保险参保达到27万人、生育保险参保达到34万人；城乡居民基本医疗保险参保550万人，财政补助标准由每人每年90元提高到120元，住院报销标准比上年提高5个百分点；推进农村饮水安全工程，解决73万农村人口饮水安全；扩大保障性住房建设，建成廉租房3.1万平方米；完成3000户历史遗留房地产权属登记；建成水库移民新村85条，解决4000户、1.8万人住房困难；新建村级敬老院120所，解决2500名农村五保户住房困难；全市农业龙头企业带动41万农户增收；改造10间乡镇卫生院；完成20户以上已通电自然村广播电视“村村通”和市区有线数字电视整体转换；建成农家书屋200个以上；建设17个农村民居地震安全示范村。

　　七、着力加强政府自身建设，提高行政执行力

　　始终把对人民负责、让人民满意作为政府工作的第一准则和第一追求，以转变职能、提高执行力为核心，不断加强自身建设，为实现科学发展、跨越发展，为争当粤西振兴发展龙头提供坚强有力保障。

　　推进法治政府建设。完善依法行政程序，严明依法行政责任，建立依法行政定期报告制度，保证各级行政机关及其工作人员严格按照法定权限和程序行使职权、履行职责。落实行政执法绩效评估奖惩办法。严格规范性文件备案审查制度，规范审查程序，健全反馈和纠正机制。加强行政复议工作，依法参加行政应诉。自觉接受人大及其常委会、政协、各民主党派与人民团体、人民群众和新闻舆论监督，积极办理人大代表议案、建议和政协委员提案，并向社会公布办理结果。

　　推进责任政府建设。健全主体明确、层级清晰、操作性强的岗位责任制，完善目标责任制和主办负责制，构筑层级之间、岗位之间的责任链条。认真落实领导干部挂钩联系重点项目、重点工作和重大事项制度，建立项目进度倒逼机制，确保任务到位、责任到位、落实到位。全面实施各级党政领导班子、领导干部和公务员年度考核评价办法，推进考核监督制度化、规范化。完善机关效能投诉中心和电子监察系统，加强行政责任和效能监察。

　　推进服务政府建设。始终把服务人民贯穿于履行政府职责的全过程，努力提高服务群众、服务基层、服务企业水平。坚持办好市长信箱、市长热线等公众平台，及时解决群众反映的重点问题。大力推进政务公开和办事公开，开通政府信息公开网。建立市、县（市、区）两级行政服务中心与审批部门、投资者“三位一体”联动机制，完善网上行政审批系统，进一步清理审批事项，简化办事程序，提高办事效率。实行政府部门审批绩效考核和通报制度。广泛运用信息化手段提高政务工作效能。

　　推进实干政府建设。进一步强化决策执行，在抓落实上见分晓、比高低、论成败。加快建立高效快捷、迅速果断的执行机制，先人一步谋事，快人一拍干事，确保每个项目、每项工作定一件、干一件、成一件。推行“工作不落实问责制”，对措施不力、行动迟缓、影响重大决策效果者，实行责任倒查追究。各级干部要扑下身子，深入实际，做到决策在一线落实，问题在一线解决，工作在一线推进。继续治理“文山会海”，切实克服形式主义和繁文缛节。

　　推进廉洁政府建设。认真贯彻执行党员领导干部廉洁从政若干准则，全面落实党风廉政建设责任制，切实履行政府廉政承诺。完善部门预算、国库集中收付、政府采购和“收支两条线”等制度，加强财政支出全过程监督，强化财政性资金投资项目和中央、省、市扩大内需专项资金审计并实行审计结果公告。建成工程招投标中心、政府采购中心、土地（产权）交易中心、住房公积金管理中心等重点部门监控系统，从源头上防治腐败。切实纠正损害群众利益的不正之风，坚决查处发生在领导机关和领导干部中的违法违纪案件。教育广大公务员严于律己、清正廉洁，老老实实做人、干干净净干事，始终保持高尚的精神追求和道德情操。

　　各位代表，东风浩荡满眼春，征程万里当奋进。我市正处在跨越发展、后发崛起的关键时期，面临难得机遇，前景无限光明。让我们在中共湛江市委的正确领导下，坚定信心，振奋精神，只争朝夕，苦干兴市，奋力争当粤西振兴发展龙头，为实现湛江在全省区域协调发展大格局中率先崛起而努力奋斗！
